# Supplementary material for: Combining DNA and protein alignments to improve genome annotation with LiftOn
Source: bioRxiv. 2024 May 17:2024.05.16.593026. Preprint. [Version 1] doi: 10.1101/2024.05.16.593026 (PMC11118573; doi:10.1101/2024.05.16.593026)
Supplement: Supplement 3 [file media-3.pdf]

Supplementary Tables of

Combining DNA and protein alignments to improve genome annotation with LiftOn

Kuan-Hao Chao[1, 2, *], Jakob M. Heinz[3], Celine Hoh[1, 2], Alan Mao[1, 2, 4], Alaina Shumate[2, 4], Mihaela Pertea[1, 2, 4], and Steven L Salzberg[1, 2, 4, 5, *]

[1]Department of Computer Science, Johns Hopkins University, Baltimore, MD 21218, USA

[2]Center for Computational Biology, Johns Hopkins University, Baltimore, MD 21218, USA

[3]Department of Biomedical Informatics, Harvard Medical School, Boston, MA 02115, USA

[4]Department of Biomedical Engineering, Johns Hopkins University, Baltimore, MD 21218, USA

[5]Department of Biostatistics, Johns Hopkins University, Baltimore, MD 21211, USA

[*]*corresponding authors:* kh.chao@cs.jhu.edu, salzberg@jhu.edu

Gene biotype	Number of genes in GRCh38	Number of genes mapped onto T2T_CHM13 by LiftOn
protein_coding	19927	20144
lncRNA	18008	18723
Pseudogene	16896	17855
miRNA	1915	2383
snoRNA	1194	1187
tRNA	453	548
V_segment	239	244
snRNA	153	192
J_segment	98	80
ncRNA	51	49
misc_RNA	42	44
C_region	21	23
antisense_RNA	19	19
other	13	13
Y_RNA	4	7
vault_RNA	4	4
scRNA	4	4
telomerase_RNA	1	1
RNase_P_RNA	1	1
RNase_MRP_RNA	1	1
**Total**	**59044**	**61522**

Table S1 The number of genes for each biotype annotated on the main chromosomes and unplaced contigs in GRCh38 and T2T-CHM13. The T2T-CHM13 annotations were generated using LiftOn by mapping the RefSeq annotations (Release 220) from GRCh38 patch 14 to T2T-CHM13 v2.0.

Gene Name	Number of extra copies
gene-LOC124905331	50
gene-TAF11L5	34
gene-USP17L11	25
gene-TSPY10	19
gene-FAM90A13	14
gene-FAM90A12	9
gene-LOC101929601	7
gene-FAM90A24, gene-TSPY4	6
gene-TSPY3	5
gene-AMY1C, gene-LOC124900996, gene-LOC124901652, gene-LOC124903857	4
gene-LOC107987371, gene-LOC124901646, gene-LOC124903544, gene-TSPY9, gene-USP17L17	3
gene-CLEC18B, gene-GOLGA6B, gene-LOC107987020, gene-LOC107987067, gene-LOC107987372, gene-LOC112268317, gene-LOC124900992, gene-LOC124901580, gene-LOC124901639, gene-LOC124901648, gene-LOC124901651, gene-LOC124901712, gene-LOC124904581, gene-LOC124905938, gene-LOC128966684, gene-NPIPB15, gene-PDPR, gene-PGA4, gene-TSPY8	2
gene-BOLA2, gene-CCL3L3, gene-CCL4L2, gene-DEFB103B, gene-DEFB104A, gene-DEFB105B, gene-DEFB106A, gene-DEFB107A, gene-DEFB4A, gene-DUSP22, gene-EIF3C, gene-FAM90A9, gene-FCGR3B, gene-FOXD4L4, gene-FRG2C, gene-GOLGA6L1, gene-GOLGA8R, gene-GPRIN2, gene-KCNJ18, gene-LGALS9C, gene-LIMS3, gene-LOC101927345, gene-LOC101929627, gene-LOC107985915, gene-LOC112268458, gene-LOC124901638, gene-LOC124901798, gene-LOC124903442, gene-LOC124903761, gene-LOC124904095, gene-LOC124905300, gene-LOC124905320, gene-LOC124907854, gene-LOC128966594, gene-MRGPRX1, gene-PRR23D2, gene-RGPD5, gene-SLX1A, gene-SPAG11B, gene-SPDYE13, gene-SPDYE9, gene-SULT1A3, gene-TPTE, gene-TSPY1, gene-USP17L7, gene-XAGE1A, gene-ZNG1C	1

Table S2. Distribution of extra copies of protein-coding genes identified by the Liftoff module in LiftOn of mapping RefSeq GCF_000001405.40-RS_2023_10 release annotations from GRCh38.p14 to T2T-CHM13 v2.0.

Gene Name	Number of extra copies
gene-LOC112268317	27
gene-LOC124903544, gene-LOC124902738, gene-IGHJ6, gene-KCNE1	1

Table S3. Distribution of extra copies of protein-coding genes identified by the miniprot module in LiftOn of mapping RefSeq GCF_000001405.40-RS_2023_10 release annotations from GRCh38.p14 to T2T-CHM13 v2.0.

	Total gene count	Protein-coding gene count			Non-coding gene count		
Reference	19,158	19,158			0		
Target (LiftOn)	19,535	*Single copy*	*Extra copy*	*Extra copy count*	*Single copy*	*Extra copy*	*Extra copy count*
		18,968	162	405	0	0	0
		19,535			0		

Table S4. Statistics for LiftOn at the gene level, as a result of mapping RefSeq MANE release v1.2 annotation from the GRCh38.p14 human genome to T2T-CHM13 v2.0 (https://github.com/marbl/CHM13).

		Total feature count	Protein-coding feature count			Non-coding feature count		
Transcript	Reference	168,451	105,328			69,250		
	Target (LiftOn)	156,173	*Single copy*	*Extra copy*	*Extra copy count*	*Single copy*	*Extra copy*	*Extra copy count*
			99,461	45	138	55,217	139	1,357
			99,599			56,574		

Table S5. The summary LiftOn statistics for lift-over results at transcript-levels, depicting the mapping from Human GRCh38 CHESS 3 v .3.0.1 (https://ccb.jhu.edu/chess/) to T2T-CHM13 v2.0 (https://github.com/marbl/CHM13)

		Total feature count	Protein-coding feature count			Non-coding feature count		
Gene	Reference	35,551	22,192			13,359		
	Target (LiftOn)	36,525	*Single copy*	*Extra copy*	*Extra copy count*	*Single copy*	*Extra copy*	*Extra copy count*
			21,507	198	840	13,195	131	654
			22,545			13,980		
Transcript	Reference	119,745	96,192			23,553		
	Target (LiftOn)	120,692	*Single copy*	*Extra copy*	*Extra copy count*	*Single copy*	*Extra copy*	*Extra copy count*
			95,169	280	986	23,284	173	800
			96,435			24,257		

Table S6 Statistics for LiftOn at both the gene and transcript levels, as a result of mapping the RefSeq GRCm39 annotation (GCF_000001635.27-RS_2023_04) from GRCm39 (C57BL/6J strain) to NOD_SCID assembly (NOD_SCID strain).

		Total feature count	Protein-coding feature count			Non-coding feature count		
Gene	Reference	11,793			9,935			1,858
	Target (LiftOn)	11,916	*Single copy*	*Extra copy*	*Extra copy count*	*Single copy*	*Extra copy*	*Extra copy count*
			9,841	54	95	1,822	23	81
					9,990			1,926
Transcript	Reference	26,617			23,471			3,146
	Target (LiftOn)	26,797	*Single copy*	*Extra copy*	*Extra copy count*	*Single copy*	*Extra copy*	*Extra copy count*
			23,287	83	144	3,078	45	160
					23,514			3,283

Table S7 Statistics for LiftOn at both the gene and transcript levels, as a result of mapping the RefSeq *Apis mellifera* annotation release 104 from Amel_HAv3.1 (GCF_003254395.2) to ASM1932182v1 assembly (GCA_019321825.1).

		Total feature count	Protein-coding feature count			Non-coding feature count		
Gene	Reference	32,008	28,738			3,270		
	Target (LiftOn)	33,450	*Single copy*	*Extra copy*	*Extra copy count*	*Single copy*	*Extra copy*	*Extra copy count*
			28,385	322	1039	3,157	108	439
			29,746			3,704		
Transcript	Reference	48,843	42,566			6,277		
	Target (LiftOn)	50,352	*Single copy*	*Extra copy*	*Extra copy count*	*Single copy*	*Extra copy*	*Extra copy count*
			42,181	346	1047	6,146	125	507
			43,574			6,778		

Table S8 Statistics for LiftOn at both the gene and transcript levels, as a result of mapping the NCBI RefSeq *Oryza sativa* Japonica Group annotation release 102 from IRGSP-1.0 (GCF_001433935.1) to ASM3414082v1 assembly (GCA_034140825.1).

<table>
<tr><th></th><th></th><th>Total feature count</th><th colspan="3">Protein-coding feature count</th><th colspan="3">Non-coding feature count</th></tr>
<tr><td rowspan="4">Gene</td><td>Reference</td><td>31,481</td><td colspan="3">27,562</td><td colspan="3">3,919</td></tr>
<tr><td rowspan="3">Target (LiftOn)</td><td rowspan="3">31,525</td><td>Single copy</td><td>Extra copy</td><td>Extra copy count</td><td>Single copy</td><td>Extra copy</td><td>Extra copy count</td></tr>
<tr><td>27,390</td><td>134</td><td>224</td><td>3,665</td><td>38</td><td>74</td></tr>
<tr><td colspan="3">27,748</td><td colspan="3">3,777</td></tr>
<tr><td rowspan="4">Transcript</td><td>Reference</td><td>52,635</td><td colspan="3">48,256</td><td colspan="3">4,379</td></tr>
<tr><td rowspan="3">Target (LiftOn)</td><td rowspan="3">52,680</td><td>Single copy</td><td>Extra copy</td><td>Extra copy count</td><td>Single copy</td><td>Extra copy</td><td>Extra copy count</td></tr>
<tr><td>48,060</td><td>146</td><td>237</td><td>4,125</td><td>38</td><td>74</td></tr>
<tr><td colspan="3">48,443</td><td colspan="3">4,237</td></tr>
</table>

Table S9 Statistics for LiftOn at both the gene and transcript levels, as a result of mapping the RefSeq TAIR10.1 annotation from TAIR10.1 (GCF_000001735.4) to Col-CEN (ASM2311539v1) assembly (GCA_023115395.1).
